# Quality of Life in Cohabitants of Patients Suffering Inflammatory Bowel Disease: A Cross-Sectional Study

**DOI:** 10.3390/ijerph19010115

**Published:** 2021-12-23

**Authors:** Manuel López-Vico, Antonio D. Sánchez-Capilla, Eduardo Redondo-Cerezo

**Affiliations:** 1 Department of Gastroenterology, “Virgen de las Nieves” University Hospital, 18014 Granada, Spain; Leunamlv@gmail.com (M.L.-V.); adasanca@gmail.com (A.D.S.-C.); 2 Instituto de Investigación Biosanitaria de Granada (ibs.Granada), 18014 Granada, Spain; 3 Endoscopy Unit, Gastroenterology Department, Virgen de las Nieves University Hospital, 18014 Granada, Spain

**Keywords:** inflammatory bowel disease, quality of life, cohabitants, IBDQ32

## Abstract

(1) Background: Inflammatory bowel disease (IBD) is a chronic inflammatory condition with a significant impact on patients’ general health perception. No studies have considered consequences of IBD on cohabitants. (2) Aims: The aims of this study were to address the influence of IBD on cohabitants’ quality of life (QoL) and the factors potentially conditioning this impact. (3) Methods: We conducted a cross-sectional study in which IBD patients and their cohabitants were invited to participate. Validated questionnaires were used to measure QoL in patients and cohabitants. Main clinical and sociodemographic variables were collected. (4) Results: A total of 56 patients and 82 cohabitants with significant QoL impairment were included. A direct association was found between Inflammatory Bowel Disease Questionnaire (IBDQ32) score in patients and the Household Members Quality of Life—Inflammatory Bowel Disease (HHMQoL-IBD). IBDQ32 was related to the number of flares in the last 12 months, number of hospital admissions and Mayo Score. (5) Conclusions: HHMQoL-IBD score was related to patients IBDQ32 score and the presence of extraintestinal disease. We identified CRP, a marker of disease activity, as a factor related to cohabitants’ quality of life, pointing to a direct relationship of patients’ disease activity and their cohabitants’ quality of life.

## 1. Introduction

Inflammatory bowel disease (IBD), including Crohn’s disease (CD) and ulcerative colitis (UC), is chronic autoimmune disease characterized by digestive symptoms, most frequently abdominal pain and diarrhea, with a relapsing clinical course and unclear etiology. A review by Molodecky et al. [1] reported that in Europe, 322 per 100,000 people suffer CD and up to 505 per 100,000 people are affected by UC, often requiring medical treatment, surgery, and hospitalizations. In addition, there has been a rise in the cost of care of IBD patients in the recent years, with a threefold higher spend in direct costs of care in IBD patients, compared to non-IBD patients, and more than twice the out-of-the-pocket costs [2].

The impact on the quality of life (QoL) of IBD patients has been well evaluated in different studies, with robust confirmation that IBD patients have a poorer quality of life compared to healthy individuals, both in adults and children, as shown in a recent systematic review and meta-analysis [3]. The impact of IBD is not limited to the patient, but it also affects the caregivers. The caregiver burden is often defined as the stress and other psychological symptoms experienced by family members and other nonprofessional caregivers by looking after individuals with mental or physical disabilities, disorders, or diseases. Several studies have evaluated the caregiver burden in IBD. A study by Parekh et al. [4] concluded that up to 44% of caregivers report high level of burden and decreased quality of life. This paper observed different factors to be related to a higher impact on caregiver burden, such as female gender, young age, or having more than one dependent. Similar results were obtained by Magro et al. [5], who found that IBD caregivers are substantially affected by anxieties and tension with the patient, with major worries related to cancer development as well as fear for family and social problems related to work. A recent review by Shukla et al. [6] reiterates these findings and addresses the lack of a specific measure for IBD caregiver burden. A study by Zand et al. [7] observes that caregiver burden is related to decreased productivity.

All these studies have evaluated the impact of IBD on caregivers, with no study directly assessing the impact of IBD on the household members’ group, cohabitants with no direct involvement in patient’s care. Due to the chronic and relapsing nature of the disease, often involving necessary treatments with risk of major adverse effects, our aims were to analyze quality of life in cohabitants of people with IBD, and to assess the potential related factors. These objectives could help to propose new approaches which include not only the patient and the main caregiver, but other household members, and take a deeper insight to the global impact of the disease.

## 2. Materials and Methods

### 2.1. Design and Study Population

We conducted a cross-sectional study that included patients with IBD and their household members. Participants were recruited consecutively from 1 June to 31 August 2021 from patients attending the IBD Unit of Virgen de las Nieves University Hospital (Granada, Spain). Participants, both patients and household members, received all the information and gave their written informed consent before completing the questionnaires. All the information was processed in accordance with current legislation to preserve the autonomy and privacy of patients and cohabitants. The protocol was approved by the Research Ethics Committee of Granada (0079-O-21).

Patients and household members were contacted by phone and were invited to participate. Inclusion criteria were as follows: Patients with definitive diagnosis of IBD at any stage and household members living in the same house over a period of 1 year or more. Exclusion criteria were as follows: Age < 18, subjects unable to complete the questionnaires, and refusal to participate. If a patient and their corresponding household members met all the inclusion criteria and none of the exclusion criteria and agreed to participate, they were included in the study.

### 2.2. Study Variables

Demographic data was obtained for both groups including age, sex, occupation, educational level, marital status, and the relationship with the patient in the case of the household members.

IBD activity was assessed by using standardized clinical indices, the partial Mayo Score and the Harvey–Bradshaw Index for ulcerative colitis and Crohn’s disease, respectively. The partial Mayo Score takes into account the stool frequency, rectal bleeding, and physician’s global assessment, and the Harvey–Bradshaw Index takes into account the patient’s wellbeing, abdominal pain, number of liquid stools, abdominal mass, and complications. Information about prior surgeries, the bowel segment resected if present, extraintestinal manifestations, articular, or extra articular ones, past and current treatments including biologics, thiopurines, calcineurin inhibitors, and others, number of relapses considered as the need of treatment escalation, use of topic or oral corticoids, number of hospitalizations since diagnosis, and laboratory data such as CPR and calprotectin was also collected.

Quality of life was assessed in the case of patients with the Inflammatory Bowel Disease Questionnaire (IBDQ), which had been previously validated in Spanish. We used the 32-item version by Masachs et al. [8]. The questions are divided into four domains as follows: Bowel symptoms (10 questions), systemic symptoms (5 questions), emotional functioning (12 questions), and social functioning (5 questions). For the household members, we used the Household Members Quality of Life—Inflammatory Bowel Disease (HHMQoL-IBD) specific questionnaire developed by Vergara et al. [9], also validated in Spanish, and this is the first time it has been used in a real population.

### 2.3. Data Analysis

Continuous variables were expressed as the mean and standard deviation (SD), and the Students’ or Wilcoxon–Mann–Whitney tests were used as appropriate. Qualitative variables were expressed as proportions and compared using the chi-square test or Fisher’s exact test, where necessary. In these cases, *p* < 0.05 was considered statistically significant. Simple linear regression was used to analyze the relationship between IBDQ32/HHMQoL-IBD and other continuous variables. ANOVA was used when the variables were qualitative. Multiple linear regression was used to estimate which variables were significantly related to HHMQoL-IBD. Dummy variables were created for categorical variables to include them in the multiple linear regression. Statistical analyses were performed with SPSS 25 (IBM Corp. Released 2017. IBM SPSS Statistics for Windows, Version 25.0. Armonk, NY, USA: IBM Corp.).

## 3. Results

Fifty-six patients and 82 cohabitants were included. Both groups showed similar sociodemographic characteristics, with no statistic differences (Table 1). Among patients, there was a similar proportion of men and women, but women were higher in proportion among cohabitants (54.9%). We included 22 patients with ulcerative colitis, 32 patients with Crohn’s disease, and 2 patients with an indeterminate colitis.

### 3.1. Patients

**Figure 1:**
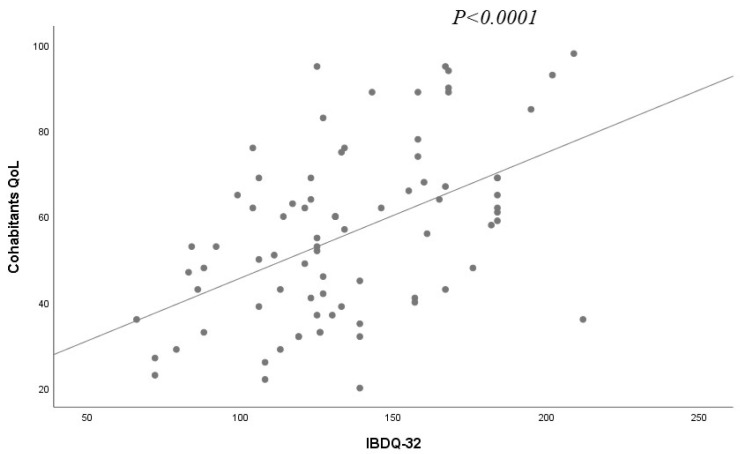
Simple linear regression for cohabitants’ QoL and IBDQ32 scores.

IBDQ32 showed a moderate mean quality of life for patients with IBD, while cohabitants showed a mean value in the HHMQoL-IBD score of 55.91. IBDQ 32 was associated with HHMQoL-IBD (*p* < 0.0001) (Figure 1), the number of flares in the last 12 months (*p* = 0.008), type of IBD (*p* = 0.007), and the Harvey–Bradshaw score (*p* = 0.001) (Table 2).

Patients with Crohn’s disease had a significantly lower IBDQ 32 score than UC patients, higher number of disease flares (*p* = 0.024) and higher need for surgery (*p* = 0.033). In these patients, calprotectin in the last control was related with IBDQ 32 (*p* = 0.017), as well as Harvey–Bradshaw index (*p* < 0.0001) (Table 3).

In a univariate linear regression analysis, the number of flares in the last 12 months (*p* = 0.007), number of hospital admissions (*p* = 0.029), and Mayo Score (*p* = 0.49) influenced IBDQ32. We also found an increasing IBDQ 32 score the longer the time from diagnosis (*p* = 0.005) (Table 2). A multivariate linear regression model showed that Mayo Score (*p* = 0.001) and the number of hospital admissions (*p* < 0.0001) had a significant impact on patients’ quality of live, measured by IBDQ 32.

We did not find differences in IBDQ32 scores regarding education level or occupation.

### 3.2. Cohabitants

**Figure 2:**
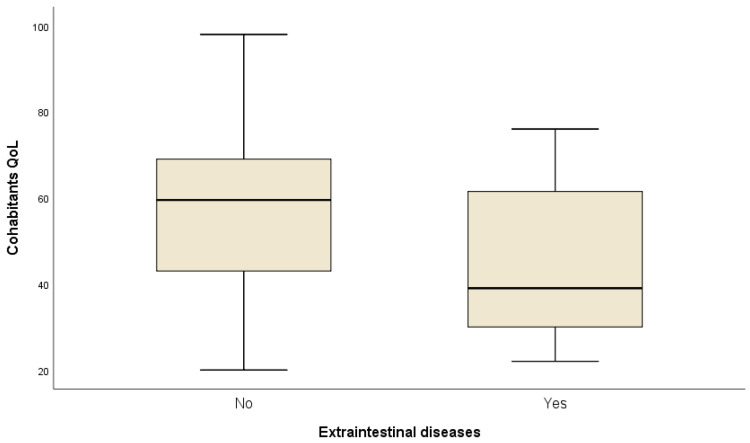
Means comparison of HHMQoL-IBD between cohabitants of patients with and without extraintestinal disease.

The univariate analysis of the cohabitants’ quality of life showed a significant association of HHMQoL-IBD and those variables: IBDQ 32 score in their relatives (*p* < 0.0001) (Figure 1), last CRP (*p* = 0.043), number of disease flares in the last year (*p* = 0.04), extraintestinal diseases (*p* = 0.01) (Figure 2), and, particularly, articular disease (*p* = 0.04) (Table 4). Among those extraintestinal diseases, we observed nine axial articular disease and two dermatologic, in the form of erythema nodosum.

**Figure 3:**
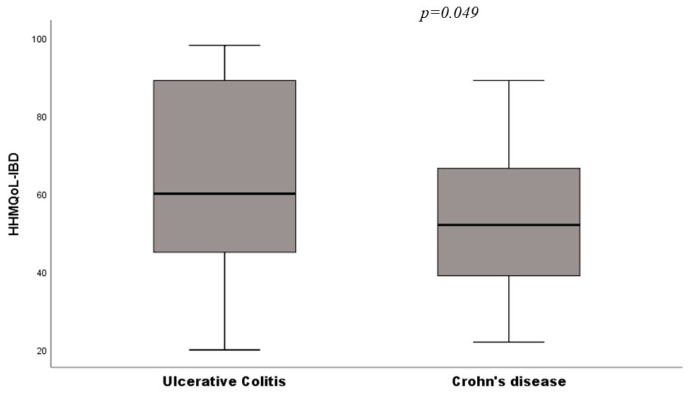
Means comparison of HHMQoL-IBD between cohabitants of patients with UC and CD.

Cohabitants of patients with Crohn’s disease had a significantly lower HHMQoL-IBD than ulcerative colitis ones (53 ± 18 vs. 62 ± 23; *p* = 0.049) (Figure 3).

Among cohabitants of patients with ulcerative colitis, patient’s CPR in the last control was related to HHMQoL-IBD score (*p* = 0.043). Cohabitant’s score was related with IBDQ32 in patients (*p* = 0.019).

Among cohabitants of patients with Crohn’s disease, patient’s CPR in the last control was related to HHMQoL-IBD score (*p* = 0.04). In this group, cohabitants score was related with IBDQ32 in patients (*p* < 0.0001). B2 phenotype and Harvey–Bradshaw index almost reach significance in their relationship with the score.

No changes were observed with increasing time from diagnosis in HHMQoL-IBD score.

**Figure 4:**
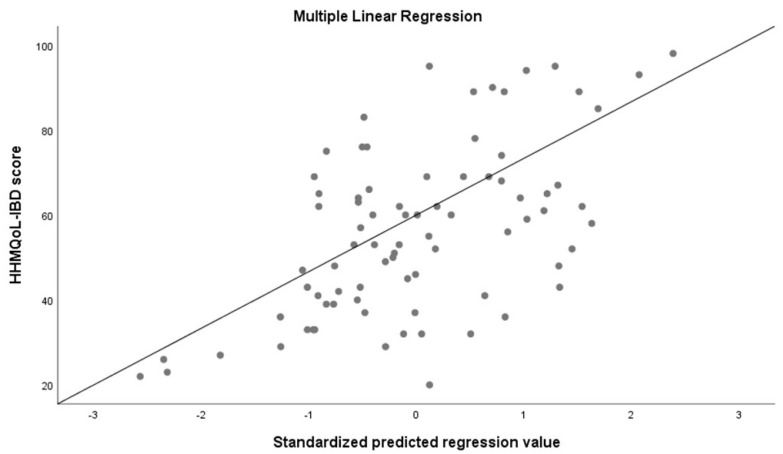
Multiple linear regression.

In a linear multiple regression analysis, IBDQ 32 score (*p* < 0.0001) in patients, as well as the presence of extraintestinal disease (*p* = 0.048), were independent predictors of cohabitants’ quality of life (Table 5). In this regard, articular disease was particularly related with a poorer quality of life among cohabitants (Figure 4).

## 4. Discussion

The impact of IBD on patients’ quality of life has been previously studied, and quality of life of the main caregivers of these patients has even been addressed [7]. Indeed, in Zand et al.’s paper [7], caregiving for IBD patients determined significant productivity decreases. Ulcerative colitis caused more burden on caregivers, and so did patients with fistulae. However, as IBS is a disease with a great clinical impact on patients’ wellbeing, it is presumable that cohabitants’ quality of life can be significantly affected by the burden of this disease, and this has not been previously studied.

Our study shows that quality of life in cohabitants is related to quality of life in IBD patients, as well as with extraintestinal manifestations, which comprise an important burden on patients’ wellbeing, and so seems the case in their cohabitants. Moreover, both in Crohn’s disease as well as in UC, HHMQoL-IBD score was related with CRP, a marker of disease activity. This finding has also been uncovered in other inflammatory and immune-mediated conditions [10,11]. The reasons for a poorer quality of life in IBD patients are multiple, but mainly related with several factors such as disruption to usual life activities, a direct impact on education, employability, and social and interpersonal functioning (sexuality, intimacy), as well as stigma and disability [3]. Poorer social and interpersonal functioning, self-perception, and self-esteem are likely to be associated with IBD-related complications, such as disease flares, extraintestinal disease, chronic changes in bowel function, etc., which, in turn, can adversely affect QoL [12]. Indeed, social and relational factors are deeply related to the burden of the disease, and this could be on the basis of the relation to disease activity, and with the invalidating extraintestinal-associated conditions, particularly articular disease.

Our data show that QoL in IBD patients is mostly related to disease severity. Indeed, hospital admissions and activity indexes were the best predictors for IBDQ32. However, we have not found a relevant influence of gender, marital status, education, or employment. A previous study on caregivers showed that apart from disease activity, gender, age, and annual income played a role on caregivers’ burden [4]. Some authors observed that UC patients’ caregivers suffered a poorer QoL than CD ones [7]. These differences are difficult to ascertain, due to the heterogeneity of populations. Indeed, we did not observe this influence of sociodemographic characteristics, and so failed to find this relationship from Zand et al. [7], but we observed worse QoL scores among cohabitants of Crohn’s disease patients. In actuality, our Crohn’s disease patients had a lower IBDQ32 score, and so did their cohabitants, not reproducing Zand’s results. A recent meta-analysis on QoL of patients with CD and UC showed worse scores for CD [13]. This is consistent with our findings, and it is quite likely that cohabitants parallel this trend. However, some authors state that disease type might not have such an influence on quality of life, but its severity, Crohn’s disease and ulcerative colitis being quite similar in this sense [13].

Previous studies have observed the strong correlation between patients’ and cohabitants’ quality of life for other immune-mediated conditions [11,14,15,16,17], as we also have observed in IBD. However, we found an increased quality of life the longer the time from diagnosis [18], that was not paralleled in cohabitants. It is probable that the previously described process of adjustment to the demands of a chronic illness determines subtle changes in a patient’s quality of life, with no influence on cohabitants, or maybe a higher sample size would provide different results. Nevertheless, QoL is a dynamic entity that can evolve with the disease and the ability of patients for adapting to and accepting their disease and symptoms [19,20]. Indeed, in other immune-mediated diseases with a worsening progressive outcome this is not the case, so a longer follow-up and reassessment should provide hints to better help patients and relatives [21].

Many factors may affect quality of life, but disease activity and disability seem to be of major importance in our research. Research in other fields with an autoimmune background and articular involvement, such as rheumatoid arthritis, has observed a direct relationship between disease activity, pain, and disability with QoL of patients [22]. These findings are quite similar to ours, supporting the hypothesis that disease activity, which is related to disability, and extraintestinal manifestations, especially articular ones, significantly affect patients’ QoL, therefore impacting on cohabitants QoL. These were also the findings of Ramos-Alejos-Pita et al. in a completely different autoimmune disease, i.e., hidradenitis suppurativa [11].

In view of our results, specific measures should be proposed to alleviate caregivers’ burden, maybe by means of support groups, psychological support, or initiatives such as schools for patients that successfully train patients to cope with several conditions.

The main limit of our research is the number of patients and cohabitants included. The topic has never been previously studied, and it is also the first time, in real clinical practice, in which the quality-of-life score in cohabitants has been applied, making the task of sample size calculation misleading and unreliable.

## 5. Conclusions

Quality of life in cohabitants of patients with inflammatory bowel disease is related to patients’ quality of life and extraintestinal conditions related to the disease. We have identified CRP, a marker of disease activity, as a factor related to cohabitants’ quality of life, pointing to a direct relationship of patients’ disease activity and their cohabitants’ quality of life. It is essential to analyze and act in the patient’s whole environment, taking into consideration not only clinical variables, which are the main ones, but also the relational and familiar conditions and impact of the disease.

## Figures and Tables

**Table 1:** Sociodemographic characteristics of patients and cohabitants.

Variables	Patients (*n* = 56)	Cohabitants (*n* = 82)
Sex		
Male	50% (28)	37 (45%)
Female	50% (28)	82 (55%)
Educational		
None	3 (5%)	8 (10%)
Primary	13 (23%)	20 (24%)
Secondary	18 (32%)	19 (23%)
University or Higher	22 (39%)	35 (43%)
Occupation		
Employed	35 (62%)	56 (69%)
Unemployed	8 (14%)	11 (13%)
Retired	13 (23%)	15 (18%)
Marital Status		
Married	42 (75%)	58 (71%)
Single	14 (25%)	24 (29%)

**Table 2:** Univariate analysis of factor potentially related with quality of life in patients.

Variables (*n*)	IBDQ 32 (Mean ± SD)	*p*
Sex Female (28) Male (28)	127 ± 34 140.6 ± 35	0.16
Age	–0.24 ± 0.33	0.46
Marital Status Single (14) Married (42)	141 ± 31 132 ± 36	0.41
Surgical treatments No (42) Yes (13)	137 ± 35 123 ± 36	0.21
Number of flares in the last year ≤2 (20) >2 (36)	145 ± 31 128 ± 36	0.008
Number of hospital admissions in the last year None (25) ≥1 (31)	134 ± 31 134 ± 39	0.99
Extraintestinal diseases No (45) Yes (11)	134 ± 36 135 ± 34	0.9
Type of IBD Ulcerative colitis (22) Crohn’s disease (32) Indeterminate colitis (2)	150.55 ± 32 121.5 ± 33 150.5 ± 36	0.007 0.009 0.006 0.8
Time from diagnosis	–0.05 ± 0.03	0.05
Ulcerative colitis extension E1 (3) E2 (4) E3 (15)	161 124 ± 42 93 ± 21	0.14
Ulcerative colitis severity S1 (15) S2 (6) S3 (1)	151 ± 32 151.5 ± 38 143	0.97
Mayo index	–3.1 ± 3.2	0.35
Crohn’s phenotype B1 (18) B2 Stenosing (8) B3 Penetrating (6)	117 ± 28 117 ± 43 140 ± 29	0.319
Perianal Crohn’s disease No (29) Yes (3)	120.5 ± 32 131 ± 49	0.6
Colonic Crohn’s disease None (19) Colonic Crohn’s (13)	126 ± 29 115 ± 39	0.29
Harvey–Bradshaw index	–5.7 ± 1.5	0.001
Extraintestinal diseases No (45) Yes (11)	134 ± 36 135 ± 34	0.89
Articular disease No (50) Yes (6)	135 ± 36 124 ± 28	0.46

**Table 3:** Comparative characteristics of Crohn’s disease and ulcerative colitis.

	Crohn’s Disease (*n* = 32)	Ulcerative Colitis (*n* = 22)	*p*
Age, Years	43 ± 13	48 ± 15	0.21
Sex			
Male	53%	46%	0.58
Female	47%	54%	
Educational			
None	6%	5%	
Primary	22%	23%	0.82
Secondary	38.50%	27%	
University or Higher	34%	46%	
Occupation			
Employee	56.30%	73%	
Unemployed	15.60%	13.60%	0.26
Retired	28.10%	13.60%	
Marital Status			
Partner	71.90%	72.30%	0.65
Single	28.10%	22.30%	
Previous surgery	34.40%	9.10%	0.033
PCR	7.15 ± 4.75	4.11 ± 3.21	0.35
Calprotectin	1121 ± 608	3909 ± 1338	0.075
HHMQoL-IBD	53 ± 18	62 ± 23	0.049
IBDQ Score	121.5 ± 33	150.5 ± 32	0.002
N° Flares since diagnosis	5.5 ± 4.4	3.8 ± 3.3	0.024
N° Hospital Admissions since diagnosis	2.1 ± 3.1	0.689 ± 0.99	0.13
Steroid response			
Steroid refractory	4%	0%	
Steroid dependency	22%	23%	0.33
Number of cohabitants			
1	59%	77%	
2	31%	9%	0.17
≥3	9%	14%	

**Table 4:** Univariate analysis of factor potentially related with quality of life in cohabitants.

Variables (*n*)	HHMQoL-IBD (Mean ± SD)	*p*
Sex		
Female (45)	56 ± 18	0.09
Male (36)	56 ± 22	
Age	–0.14 ± 0.13	0.68
Marital Status		
Single (24)	57 ±19	0.65
Married (58)	55 ± 20	
IBDQ 32 (patients)	0.28 ± 0.05	<0.0001
Surgical treatments		
No (59)	56 ± 21	0.7
Yes (21)	55 ± 17	
Number of flares in the last year		
≤2 (25)	58 ± 22	0.52
>2 (56)	55 ± 19	
Number of hospital admissions in the last year		
None (33)		
≥1 (48)	53 ± 22	0.2
	58 ± 18	
Extraintestinal diseases		
No (66)	59 ± 19	0.01
Yes (15)	44 ± 19	
Type of IBD		
Ulcerative colitis (29)	62 ± 23	0.15
Crohn’s disease (59)	53 ± 8	
Indeterminate colitis (2)	50.5 ± 3.5	
Ulcerative colitis extension		
E1 (3)	59 ± 27	
E2 (7)	74 ± 32	0.27
E3 (19)	58 ± 17	
Ulcerative colitis severity		
S1 (19)	25 ± 6	0.2
S2 (9)	52 ± 12	
S3 (1)		
Mayo index	–2.68 ± 1-18	0.16
Crohn’s phenotype		
B1 (27)	52 ± 18	
B2 Stenosing (10)	44 ± 20	0.075
B3 Penetrating (13)	61 ± 13	
Harvey–Bradshaw index	–1.46 ± 0.75	0.056
Perianal Crohn’s disease		
No (42)	51.5 ± 19	0.12
Yes (8)	59.5 ± 12	
Colonic Crohn’s disease		
None (34)	55 ± 17	0.12
Yes (16)	47 ± 19	
Articular disease		
No (52)	57.5 ± 20	0.04
Yes (9)	43 ± 17	

**Table 5:** Association between factors and HHMQoL-IBD score using multiple linear regression analysis.

Variables	Standardized Coefficients (Beta)	*p*
Sex		
Male	−0.069	0.51
Female	Ref	
Age	−0.038	0.78
Marital Status		
Married	−0.098	0.47
Single	Ref	
IBDQ 32 (patients)	0.064	<0.0001
Time from diagnosis	−0.018	0.867
Education		
University	0.015	0.894
Other (none, primary, secondary)	Ref.	
Patient’s occupation		
Active	−0.087	0.431
Unemployed	Ref.	
Last CRP	−0.084	0.433
Number of cohabitants	0.086	0.452
Extraintestinal diseases	−0.21	0.048

## Data Availability

Full study protocol and database can be requested from the corresponding author.
